# The adductor pollicis muscle thickness is not associated with physical function, lean mass, and nutritional status in patients on maintenance hemodialysis

**DOI:** 10.3389/fnut.2024.1502309

**Published:** 2025-01-22

**Authors:** Débora Moreira Morais, Isadora Cordeiro Trombim, Cassiana Regina de Góes, Barbara Perez Vogt

**Affiliations:** ^1^Faculty of Medicine, Federal University of Uberlandia (UFU), Uberlandia, Brazil; ^2^Institute of Biological and Health Sciences, Federal University of Viçosa, Campus Rio Paranaíba, Rio Paranaíba, Brazil

**Keywords:** chronic kidney failure, dialysis, nutritional assessment, physical functioning, thumb adductor muscle

## Abstract

**Background:**

The adductor pollicis muscle thickness (APMT) may be associated with the muscle strength in patients on hemodialysis. However, the association of APMT with other physical function assessment tests has not yet been tested. Moreover, because it is considered a good nutritional indicator and not influenced by fluid overload, the APMT may be associated with the muscle mass and nutritional status of these patients. Therefore, the objective was to assess the association of APMT with physical function, muscle mass and nutritional status in patients on hemodialysis.

**Methods:**

The APMT was measured using a skinfold caliper between pollicis finger and index finger. Physical function was evaluated by handgrip strength (HGS), Short Physical Performance Battery (SPPB), the sit-to-stand test, gait speed test, and timed up and go (TUG). Appendicular muscle mass index (AMMI) was estimated using bioelectrical impedance. The nutritional status was evaluated by the Malnutrition Inflammation Score (MIS).

**Results:**

Fifty-one patients were included, 60.8% men, mean age 58.4 ± 12.6 years. There were no significant correlations of APMT with physical function, muscle mass and nutritional status. Values of APMT were not different between the groups according to adequate physical function or muscle mass. In the multiple linear regression analysis adjusted for sex, age and diabetes, APMT was not significantly associated with physical function tests, as HGS (*β* = 0.101; *p* = 0.778), gait speed (*β* = −0.014; *p* = 0.180), SPPB (*β* = −0.054; *p* = 0.590), TUG (*β* = 0.202; *p* = 0.109), lean mass AMMI (*β* = 0.058; *p* = 0.147).

**Conclusion:**

There were no associations of APMT with physical function, muscle mass and nutritional status in patients on hemodialysis. We suggest APMT should not be used in physical function and nutritional assessments of these patients.

## 1 Introduction

Individuals with chronic kidney disease (CKD) undergoing hemodialysis are at an increased risk of protein depletion and impaired physical function. The factors contributing to this include imbalance between muscle catabolism and anabolism, loss of nutrients and amino acids for the dialysate, and increase of energy expenditure during the dialysis procedure, insulin and anabolic hormones resistance, increased oxidative stress and low-grade chronic inflammation (1). Thus, both muscle mass and physical function should be routinely monitored, as their reduction are significant contributors to quality of life decrease, morbidity, and mortality (2). Simple tests such as handgrip strength (HGS), gait speed test, sit-to-stand test, short physical performance battery (SPPB) and timed up and go (TUG) can be used to assess physical function (3). Accurate and early diagnosis of decrease of muscle function and muscle mass are crucial in these patients, since it is important to initiate interventions to prevent the progression or delay these complications.

The adductor pollicis muscle (APM) is an unique muscle of the hand, lying in the deepest muscular plane of the palm (4). APM thickness (APMT) is a simple, low-cost, accessible and non-invasive anthropometric measurement. Because of its anatomic characteristic and localization in the hand, APM is the only muscle that can be directly measured with a skinfold caliper (5). Additionally, APMT is minimally affected by body fat (6) and fluid overload, which is a significant issue in the anthropometric assessment of patients with CKD and on hemodialysis (2). This parameter can be considered an useful nutritional parameter (7, 8) and has already been tested as a marker of muscle mass, nutritional risk and nutritional status in general (9, 10) and clinical populations (11–15). However, the results are still contradictory.

Some studies have shown a weak association between lean mass and APMT in postmenopausal women, as well as a weak correlation between APMT and both muscle mass and lean mass in kidney transplant patients (13). On the other hand, Ishimoto et al. (16) showed that APMT can be useful for diagnosing low ALMI in older women undergoing outpatient rehabilitation. Pereira et al. (17) found that APMT was able to predict HGS and would be a good nutritional marker in patients on hemodialysis. Therefore, APMT may be associated with muscle strength in this population. However, the association of APMT with other physical function assessments have not yet been tested in patients on hemodialysis. Therefore, the aim of this study was to evaluate the association of APMT with physical function (muscle strength and physical performance), lean mass and nutritional status of patients on maintenance hemodialysis.

## 2 Methods

### 2.1 Study design and participants

This cross-sectional study enrolled a convenience sample of patients aged ≥18 years, on maintenance hemodialysis for at least three months at the Clinics Hospital of the Federal University of Uberlandia, Uberlandia, Brazil. The assessment of APMT, physical function, and nutritional status were all conducted on the same dialysis day. Individuals with physical disabilities or limb amputations that hindered any of the assessments of physical performance and muscular strength, or any catabolic pathology, such as neoplasia, advanced liver disease, heart disease or chronic obstructive pulmonary disease, sepsis, that influences body composition, physical function or APMT were excluded. Patients who were using medications that influence body composition, such as corticosteroids and antiretroviral therapy, were also excluded.

The study protocol was approved by Research Ethics Committee of the Federal University of Uberlandia (CAAE: 59193822.3.0000.5152; protocol number: 5.591.566) and it was in accordance with the ethical standards of the Helsinki Declaration. All participants were instructed about the assessments and signed the consent form.

Demographic, clinical and laboratory data were collected from the medical records. Laboratory tests performed on the patient’s routine in the same month of the assessments, such as serum creatinine, urea, albumin, and C-reactive protein, were considered.

The body weight was measured after the hemodialysis session, as this is the moment the patients are closest to their dry weight. The body mass index (BMI) was calculated by dividing the body weight by the squared height.

### 2.2 Adductor pollicis muscle thickness assessment

The APMT measurement was conducted on the opposite hand of the arteriovenous fistula arm or on the dominant hand if there was no fistula (17), before the hemodialysis session. The patient was seated, with the hand relaxed and resting on the knee, with the arm on the thigh and the elbow flexed at 90°. The muscle was measured at the imaginary vertex of the triangle formed by the thumb and index finger using a Lange skinfold caliper (Cambridge Scientific Industries, Inc., Cambridge, MD) (7). This measurement was repeated three times by the same evaluator, and the average of these values was used for the analysis (7).

### 2.3 Lean mass assessment

Appendicular lean mass (ALM) was evaluated using bioelectrical impedance (Biodynamics® model 450) 20 to 30 min after the end of the hemodialysis session. Reactance and resistance were obtained to estimate ALM by the equation proposed by Sergi et al. (18). The appendicular lean mass index (AMMI) was calculated by dividing the estimated ALM by squared height. The cutoff values to classify adequate or inadequate muscle mass were proposed in the revised Sarcopenia Consensus by the European Working Group on Sarcopenia in Older People (EWGSOP 2) (19).

### 2.4 Physical function assessment

Physical function was evaluated before the dialysis session, by the following tests: HGS, sit-to-stand test, 4 m gait speed test, SPPB and TUG. The cutoff values proposed in the EWGSOP 2 (19) were considered to classify adequate or inadequate physical function.

Muscle strength was assessed by HGS and sit-to-stand test. To evaluate HGS, a Jamar hydraulic dynamometer was used. The participant was seated with the elbow of the nonfistula arm flexed at 90°. Participants who do not have a fistula were evaluated in the dominant arm. The procedure was repeated three times, and the highest value was adopted for analysis (20). Adequate HGS was considered ≥27 kg and ≥ 16 kg for men and women, respectively (19). In the sit-to-stand test, the time taken to sit down and stand up from the chair five times in a row without using the arms was considered (21). Adequate muscle strength was considered ≤15 s for completion (19).

Physical performance was assessed by 4 m gait speed test, SPPB, and TUG. In the 4 m gait speed test, the patient walked a distance of four meters while the time was recorded. The gait speed was calculated by dividing the distance by the time (21). The cutoff for adequate performance was ≥0.8 m/s (19). The SPPB is a composite score that combines the results of the balance test, gait speed, and the sit-to-stand test. In the balance test, the patient remained on three different positions for 10 s each: feet side by side, semi-tandem and tandem position (21). The gait speed and sit-to-stand tests procedures were described above. Adequate performance was considered ≥8 (19). For TUG, the time the patient took to stand up from a chair, walk three meters, turn 180°, walk back to the chair, and sit down was recorded. Participants were instructed to not to use their hands when getting up or sitting down (22). The cutoff for adequate performance was ≤20 s (19).

### 2.5 Nutritional status assessment

To evaluate the nutritional status, the Malnutrition Inflammation Score (MIS) was used. This questionnaire consists of 10 questions, and each question is scored from zero to three points, with higher scores indicating worse nutritional status (23).

### 2.6 Statistical analysis

Date were expressed as mean and standard deviation or median and quartile, according to the distribution of the variables. Frequencies were expressed as percentages. Individuals were grouped according to the results of the physical function assessment and ALMI, and the APMT was compared using the Student’s *t*-test.

The Pearson or Spearman coefficients were used to assess the correlation between APMT and parameters of physical function, muscle mass, and nutritional status. Multiple linear regression models were constructed with physical function, muscle mass, or nutritional status parameters as the dependent variables, and APMT as the independent variable. Adjustments were made for age, gender, and diabetes. Statistical significance was defined as *p* < 0.05. All analyses were performed using IBM SPSS Statistics for Windows, Version 20.0 (Armonk, NY: IBM Corp).

## 3 Results

Fifty-one patients were included, with a mean age of 58 years, and the majority were men (60.8%). The most prevalent underlying kidney disease was hypertensive nephrosclerosis (*n* = 21, 41.2%), followed by undefined causes (*n* = 14, 27.5%), diabetic nephropathy (*n* = 3, 5.9%), autosomal recessive polycystic kidney disease (*n* = 2, 3.9%), and other causes (*n* = 11, 21.7%), such as benign prostatic hyperplasia, chronic glomerulopathy, multiple myeloma, and lupus nephritis. The other characteristics of the enrolled patients are shown in Table 1.

**Table 1:** Demographic and clinical characteristics of the studied population.

Characteristics	*n* = 51
Age (years)	58.4 ± 12.6
Men [*n* (%)]	31 (60.8)
Diabetes [*n* (%)]	20 (39.2)
Dialysis vintage (months)	23 (5–64)
Body mass index (kg/m^2^)	26.6 ± 6.0
Creatinine (mg/dL)	9.0 ± 3.6
Urea (mg/dL)	112 (72–125)
Albumin (g/dL)	4 (3.5–4.0)
C-reactive protein (mg/dl)	0.41 (0.14–1.0)
Kt/V	1.29 ± 0.28
APMT (mm)	12.4 ± 3.0
Handgrip strength (kgf)	28 (20–33)
Sit-to-stand test (s)	14.5 (12.7–16.9)
Gait speed (m/s)	0.88 ± 0.24
SPPB	10 (8–11)
Timed up and go (s)	11 (9.5–15.5)
ALM (kg)	18.40 ± 3.31
ALMI (kg/m^2^)	6.76 ± 0.87
Malnutrition inflammation score	3 (2–5)

APMT, adductor pollicis muscle thickness; ALM, appendicular lean mass; ALMI, appendicular lean mass index; SPPB, short physical performance battery.

There was no significant difference of APMT values between the genders (males: 13.0 ± 3.3 mm; females: 11.6 ± 2.3 mm; *p* = 0.107). However, HGS and ALM showed significant differences between the genders (*p* < 0.001). Median HGS was 30 (26–38) kg for males and 20 (16–28) kg for females. The mean ALM was 19.77 ± 3.14 kg for males and 16.28 ± 2.36 kg for females. Nonetheless, when comparing the ALMI between genders (males: 6.90 ± 0.79 kg/m^2^ and females: 6.55 ± 0.96 kg/m^2^), no significant difference was observed (*p* = 0.167). The other physical function parameters were not significant different between the genders.

ALMI was inadequate in 37.3% of patients. Regarding physical performance, 64.7, 29.4, and 5.8% of patients had inadequate gait speed, SPPB, and TUG, respectively. Regarding the HGS, 23.5% of patients had values below the sex-specific cutoff point. Two patients were unable to complete the sit-to-stand test. Among the 49 patients who performed the test, 42.8% had results of inadequate muscle strength. APMT was compared between groups according to the cutoffs for each physical function test and ALMI, and no significant differences were found (Table 2).

**Table 2:** Comparison of adductor pollicis muscle thickness between patient groups according to physical function and lean mass variables (*n* = 51).

Physical function or lean mass parameters according to EWGSOP cutoffs	APMT (mm)	*p*
Gait speed ≤0.8 m/s (*n* = 33)	12.68 ± 3.09	0.46
Gait speed >0.8 m/s (*n* = 18)	12.03 ± 2.80	
SPPB >8 points (*n* = 36)	12.46 ± 3.12	0.98
SPPB ≤8 points (*n* = 15)	12.44 ± 2.73	
Timed up and go <20s (*n* = 48)	12.45 ± 2.96	0.95
Timed up and go ≥20s (*n* = 3)	12.55 ± 4.07	
HGS ≥ 27 kg for men and ≥ 16 kg for women (*n* = 39)	12.58 ± 3.13	0.59
HGS < 27 kg for men and < 16 kg for women (*n* = 12)	12.05 ± 2.51	
Sit to stand test ≤15 s (*n* = 28)	12.54 ± 3.30	0.95
Sit to stand test >15 s (*n* = 21)	12.49 ± 2.60	
ALMI >7.0 e 5.5 (*n* = 32)	12.63 ± 3.02	0.59
ALMI <7.0 e 5.5 (*n* = 19)	12.16 ± 2.98	

EWGSOP, European working group on Sarcopenia in older people; APMT, adductor pollicis muscle thickness; SPPB, short physical performance battery; HGS, handgrip strength; ALMI, appendicular lean mass index.

**Figure 1:**
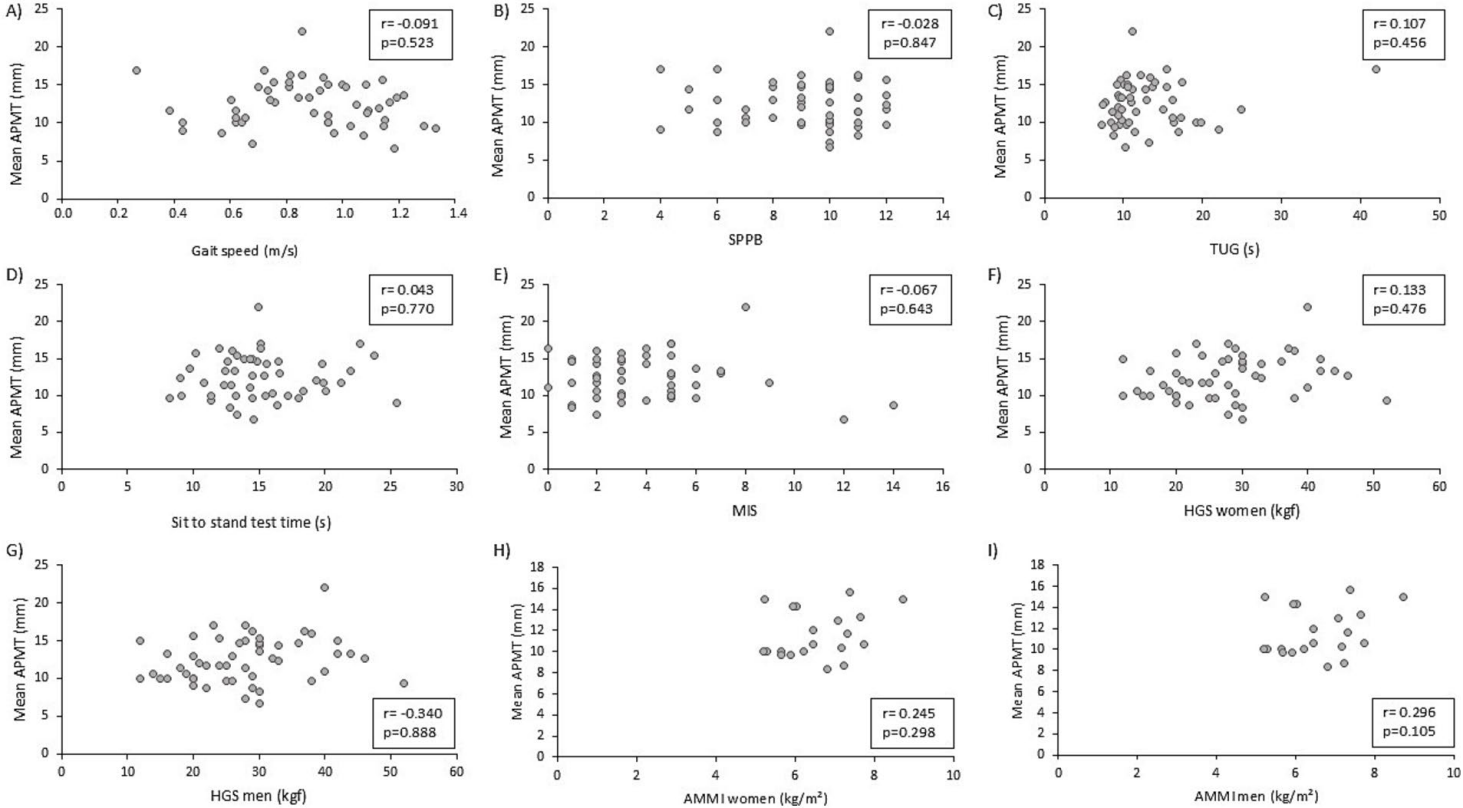
Correlation of adductor pollicis muscle thickness (APMT) with physical function markers, nutritional status, and muscle mass. **(A)** APMT and gait speed; **(B)** APMT and short physical performance battery; **(C)** APMT and timed up and go; **(D)** APMT and sit-to-stand test; **(E)** APMT and malnutrition inflammation score; **(F)** APMT and handgrip strength (female); **(G)** APMT and handgrip strength (male); **(H)** APMT and appendicular muscle mass index (female); **(I)** APMT and appendicular muscle mass index (male).

There was no significant correlation between APMT and markers of physical function, such as gait speed, SBBP, TUG, sit-to-stand test, and nutritional status (MIS) (Figures 1A–E). HGS was not significantly correlated (*r* = 0.222; *p* = 0.117). APMT was significantly and positively correlated with ALM and ALMI in the total sample (*r* = 0.304; *p* = 0.030 and *r* = 0.289; *p* = 0.039). The correlations of APMT with HGS and ALMI were also tested separately for each gender due to the different values between males and females. No significant correlations were found between these variables (Figures 1F–I).

In the multiple linear regression analysis, APMT was not significantly associated with variables of physical function or muscle mass (Table 3). The models including the sit-to-stand test as the dependent variable were not significant, as well as the models that included MIS as the dependent variable. Although other models that included different parameters of physical function and muscle mass were significant, the association between APMT and the dependent variables was not significant. The adjustments for sex, age, and diabetes increased the models predictability (Supplementary Table S1).

**Table 3:** Association of adductor pollicis muscle thickness with physical function, lean mass, and nutritional status (*n* = 51).

Dependent variable	Model	Model *R*^2^	Model *p*-value	*β* (95% CI)	*p*-value
Handgrip strength	Crude	0.050	0.115	0.681 (−0.172–1.534)	0.115
	1	0.437	<0.001	0.094 (−0.609–0.797)	0.789
	2	0.438	<0.001	0.101 (−0.615–0.816)	0.778
Sit-to-stand test	Crude	0.002	0.767	0.057 (−0.325–0.438)	0.767
	1	0.058	0.435	0.126 (−0.275–0.528)	0.529
	2	0.071	0.505	0.100 (−0.309–0.509)	0.625
Gait speed	Crude	0.008	0.523	−0.008 (−0.031–0.016)	0.523
	1	0.286	0.001	−0.160 (−0.037–0.005)	0.133
	2	0.320	0.001	−0.014 (−0.035–0.007)	0.180
Short physical performance battery	Crude	0.000	0.910	−0.012 (−0.217–0.194)	0.910
	1	0.153	0.048	−0.072 (−0.274–0.130)	0.478
	2	0.194	0.038	−0.054 (−0.255–0.147)	0.590
Timed up and go	Crude	0.000	0.966	0.007 (−0.317–0.330)	0.966
	1	0.473	<0.001	0.219 (−0.036–0.474)	0.091
	2	0.510	<0.001	0.202 (−0.047–0.452)	0.109
Appendicular lean mass index	Crude	0.091	0.032	0.088 (0.008–0.167)	**0.032**
	1	0.128	0.089	0.183 (−0.088–0.455)	0.181
	2	0.244	0.011	0.058 (−0.021–0.137)	0.147
Appendicular lean mass	Crude	0.103	0.022	0.355 (0.054–0.657)	**0.022**
	1	0.366	<0.001	0.183 (−0.088–0.455)	0.181
	2	0.405	<0.001	0.156 (−0.111–0.424)	0.246
Malnutrition inflammation score	Crude	0.019	0.351	0.098 (−0.111–0.306)	0.351
	1	0.051	0.501	0.137 (−0.087–0.362)	0.225
	2	0.073	0.494	0.149 (−0.077–0.375)	0.190

CI, confidence interval.

Model 1 was adjusted for sex and age. Model 2 was adjusted for sex, age and diabetes. *P*-value in bold corresponds to *p* < 0.05.

## 4 Discussion

Although APMT was considered a good marker of nutritional status in hemodialysis patients (8) and has been associated with muscle strength measured through HGS (17) by other studies, our study did not find a significant association between APMT and parameters of muscle strength, physical performance, muscle mass, and nutritional status in these sample of patients on maintenance hemodialysis.

APMT would be a good alternative marker of muscle mass, as it is a simple, low-cost method and is not significantly influenced by overhydration, which is likely to be altered hemodialysis patients. Due to the substantial variation in fluid balance among these patients, traditional anthropometric measures are limited in accurately assessing body composition in patients with CKD on dialysis (2).

Contrary to our findings, Pereira et al. (17) reported a positive correlation between APMT and HGS. It is not specified whether APMT measurements were performed before or after hemodialysis sessions in their study. In our study, the measurements were taken prior to the hemodialysis session, at the same time of physical function assessments, and there is no evidence whether APMT is influenced by the patient’s state of hyperhydration. Both studies did not find a significant correlation between APMT and nutritional status, which was assessed using the Subjective Global Assessment (SGA) in the study by Pereira et al. (17), and by the Malnutrition-Inflammation Score (MIS) in our study. SGA and MIS are similar in most of their assessment criteria.

Although the reduction of APMT may show the working life decrease as a consequence to a clinical condition (24), such as CKD, we did not find a significant association between physical function with APMT. One explanation for the lack of association between physical function and nutritional status with APMT is that the loss of muscle mass and muscle strength occur at different rates, as muscle strength decreases earlier and at a faster rate than muscle mass reduction (25). It is possible that the parameters of physical function evaluated in our study were reduced before any noticeable reduction in muscle quantity, which could also reflect a decline in nutritional status. Consequently, the muscle thickness measured in our study might not reflect these changes early.

Furthermore, the different tests of physical function performed in our study evaluate different aspects. For example, although both the sit-stand test and HGS assess muscle strength, they target different limbs. It would be more plausible for APMT to correlate with HGS since both are measured in the same limb. HGS measures isometric muscle strength, therefore, no movement of muscle contraction is involved. In contrast, the sit-to-stand test assesses muscle endurance (3) and requires a combination of balance control, speed strength, and muscle power of lower extremity (26). Additionally, gait speed, SPPB, and TUG evaluate other aspects such as balance, agility, and cardiorespiratory function (26), which may be less associated with muscle mass.

All the physical function tests and muscle mass assessments performed in our study were recommended by the EWGSOP 2 (19) to diagnose sarcopenia. Since APMT would be associated with both muscle mass and muscle function, the association of APMT with sarcopenia was already verified by some studies. Vaez et al. (27) showed APMT was significantly correlated with calf circumference, handgrip strength and gait speed in the elderly. They also considered APMT as a good tool to predict sarcopenia using a receiver operating characteristic (ROC) curve. Nevertheless, the APMT values proposed by Vaez et al. (27) as cutoffs for sarcopenia diagnosis are much higher than the values obtained in our study (all: 17.63 mm; female: 17.63 mm; male: 18.51 mm). On the other hand, Avancini et al. (28) did not find an association between APMT and the risk of sarcopenia assessed by the SARC-CalF questionnaire in patients with hematological cancer. Therefore, the association of APMT with sarcopenia should be further explored in different populations.

Since APMT measurements were performed by two different evaluators in our study, one of the limitations is the potential interobserver variation, as well as for all other anthropometric measurements. To minimize this limitation, we implemented standardization and training protocols prior to data collection. Moreover, APMT presents a well defined anatomic referential, which make it more easily reproducible by independent observers. Additionally, other limitation that should be recognized is the use of a convenience small sample of patients from a single center, which may limit the generalization of the results. Nevertheless, many physical function parameters were tested, and none of them were significantly associated. At last, this study was not initially designed to investigate the association of APMT with physical function, muscle mass or nutritional status. Therefore, we highlight the need for studies with greater methodological control, including measurements performed by a single evaluator and multicenter and more representative samples.

In conclusion, there was no association between APMT and physical function, muscle mass or nutritional status in this sample of patients on maintenance hemodialysis. Therefore, APMT does not appear to be a reliable anthropometric measurement as a physical function, muscle mass or nutritional marker for patients with CKD on hemodialysis.

## Data Availability

The raw data supporting the conclusions of this article will be made available by the authors, without undue reservation.
