# Supplementary material for: The adductor pollicis muscle thickness is not associated with physical function, lean mass, and nutritional status in patients on maintenance hemodialysis
Source: Front Nutr. 2025 Jan 22;11:1502309. doi: 10.3389/fnut.2024.1502309 (PMC11796473; doi:10.3389/fnut.2024.1502309)
Supplement: Supplementary file 1 [file Table_1.DOCX]

**Supplementary material**

**Table S1:** Association of Adductor Pollicis Muscle Thickness with physical function and muscle mass (n=51).

| Dependent variable | Model | Independent variable | β (95% CI) | p-value |
| --- | --- | --- | --- | --- |
| Handgrip strength | 1 | APMT | 0.094 (-0.609 – 0.797) | 0.789 |
|  |  | Sex (male) | 12.075 (7.650 – 16.500) | <0.001 |
|  |  | Age | -0.271 (-0.440 – -0.102) | 0.002 |
|  | 2 | APMT | 0.101 (-0.615 – 0.816) | 0.778 |
|  |  | Sex (male) | 12.150 (7.576 - 16.723) | <0.001 |
|  |  | Age | -0.267 (-0.445 – -0.090) | 0.004 |
|  |  | Diabetes | -0.358 (-4.864 - 4.149) | 0.874 |
| Sit-to-stand test | 1 | APMT | 0.126 (-0.275 – 0.528) | 0.529 |
|  |  | Sex (male) | -0.788 (-3.346 – 1.771) | 0.538 |
|  |  | Age | 0.078 (-0.018 – 0.173) | 0.108 |
|  | 2 | APMT | 0.100 (-0.309 – 0.509) | 0.625 |
|  |  | Sex (male) | -0.939 (-3.539 – 1.661) | 0.471 |
|  |  | Age | 0.066 (-0.034 – 0.167) | 0.190 |
|  |  | Diabetes | 0.990 (-1.555 – 3.535) | 0.437 |
| Gait Speed | 1 | APMT | -0.160 (-0.037 – 0.005) | 0.133 |
|  |  | Sex (male) | 0.090 (-0.044 – 0.224) | 0.182 |
|  |  | Age | -0.110 (-0.016 – -0.006) | <0.001 |
|  | 2 | APMT | -0.014 (-0.035 – 0.007) | 0.180 |
|  |  | Sex (male) | 0.111 (-0.024 – 0.247) | 0.105 |
|  |  | Age | -0.010 (-0.015 – -0.005) | <0.001 |
|  |  | Diabetes | -0.100 (-0.234 – 0.033) | 0.137 |
| Short physical performance battery | 1 | APMT | -0.072 (-0.274 – 0.130) | 0.478 |
|  |  | Sex (male) | 0.693 (-0.580 – 1.966) | 0.279 |
|  |  | Age | -0.070 (-0.119 – -0.022) | 0.005 |
|  | 2 | APMT | -0.054 (-0.255 – 0.147) | 0.590 |
|  |  | Sex (male) | 0.894 (-0.390 - 2.179) | 0.168 |
|  |  | Age | -0.060 (-0.110 – -0.010) | 0.019 |
|  |  | Diabetes | -0.956 (-2.222 – 0.309) | 0.135 |
| Timed up and go | 1 | APMT | 0.219 (-0.036 – 0.474) | 0.091 |
|  |  | Sex (male) | -2.243 (-3.802 – - 0.684) | 0.006 |
|  |  | Age | 0.183 (0.124 - 0.243) | <0.001 |
|  | 2 | APMT | 0.202 (-0.047 – 0.452) | 0.109 |
|  |  | Sex (male) | -2.542 (-4.099 – -0.985) | 0.002 |
|  |  | Age | 0.170 (0.110 – 0.230) | <0.001 |
|  |  | Diabetes | 1.383 (-0.157 – 2.923) | 0.077 |
| Appendicular lean mass index | 1 | APMT | 0.183 (-0.088 – 0.455) | 0.181 |
|  |  | Sex (male) | 0.328 (-0.197 – 0.854) | 0.215 |
|  |  | Age | -0.010 (-0.031 – 0.010) | 0.299 |
|  | 2 | APMT | 0.058 (-0.021 – 0.137) | 0.147 |
|  |  | Sex (male) | 0.190 (-0.316 – 0.696) | 0.454 |
|  |  | Age | -0.017 (-0.037 – 0.002) | 0.080 |
|  |  | Diabetes | 0.658 (0.159 – 1.156) | 0.011 |
| Appendicular lean mass | 1 | APMT | 0.183 (-0.088 - 0.455) | 0.181 |
|  |  | Sex (male) | 3.721 (2.012 – 5.430) | <0.001 |
|  |  | Age | -0.065 (-0.130 – 0.000) | 0.051 |
|  | 2 | APMT | 0.156 (-0.111 – 0.424) | 0.246 |
|  |  | Sex (male) | 3.415 (1.704 – 5.127) | <0.001 |
|  |  | Age | -0.081 (-0.147 - -0.014) | 0.019 |
|  |  | Diabetes | 1.455 (-0.231 – 3.141) | 0.089 |
| Malnutrition inflammation score | 1 | APMT | 0.137 (-0.087 – 0.362) | 0.225 |
|  |  | Sex (male) | -0.533 (-1.883 – 0.817) | 0.430 |
|  |  | Age | 0.029 (-0.021 – 0.078) | 0.256 |
|  | 2 | APMT | 0.149 (-0.077 – 0.375) | 0.190 |
|  |  | Sex (male) | -0.390 (-1.769 - 0.989) | 0.571 |
|  |  | Age | 0.036 (-0.016 – 0.088) | 0.172 |
|  |  | Diabetes | -0.685 (-2.032 – 0.661) | 0.311 |

Abbreviation: CI, confidence interval; APMT: Adductor Pollicis Muscle Thickness
